# Effect of the use of remineralization agents before resin infiltration on the treatment of initial enamel lesions: an in-vitro study

**DOI:** 10.1186/s12903-024-04523-z

**Published:** 2024-07-30

**Authors:** Bengü Doğu Kaya, Aybike Manav Özen, Pınar Yılmaz Atalı, Ayça Sarıalioğlu Güngör, Evrim Dalkılıç, Elif Alkan, Dilek Tağtekin, Cafer Türkmen

**Affiliations:** 1 Faculty of Dentistry, Department of Restorative Dentistry, Marmara University, Istanbul, Turkey; 2 Faculty of Dentistry, Department of Restorative Dentistry, İstanbul Galata University, Istanbul, Turkey; 3 Faculty of Dentistry, Department of Restorative Dentistry, Bezmialem Vakıf University, Istanbul, Turkey

**Keywords:** Enamel lesion, Remineralization, Resin infiltration, OCT, SEM/EDX

## Abstract

**Aim:**

This study aimed to evaluate the effect of the use of remineralization agents before the application of resin infiltration on the treatment of initial enamel lesions.

**Materials and methods:**

Eighty buccal enamel samples were prepared from human molars, and artificial initial lesions were formed after 96 h of incubation with a demineralizing solution. The samples were randomly divided into 8 groups (*n* = 10) including a remineralizing agent (Tooth Mousse, Medical Mineral Gel, Remin Pro), resin infiltration (ICON), and a combined treatment of both. Remineralizing agents were applied in pH cycle for 7 days. Baseline, demineralization, and after-treatment fluorescence (FluoreCam and DIAGNOdent Pen), surface microhardness (HMV-2T), surface roughness (M300C), OCT (Maestro-2) and ultrasonic system (Novascope 4500) data were obtained for all groups. The sample surfaces were examined under SEM/EDX (SU3500) at x1000. Data were statistically analyzed using the Two-Way Robust ANOVA and Bonferroni tests (*p* < 0.05).

**Results:**

There was no statistically significant difference between the groups for microhardness, roughness, OCT, DIAGNOdent Pen, ultrasound, and FluoreCam size/intensity values (*p* = 0.582; *p* = 0.963; *p* = 0.884; *p* = 0.923; *p* = 0.051; *p* = 0.268; *p* = 0.793 respectively). The effect of the treatment procedure showed a significant difference (*p* < 0.001), except for the roughness values (*p* = 0.984). The lowest Calcium (Ca) ratio (%atomic) was observed in the RI group in the EDX analysis.

**Conclusion:**

Remineralizing agents and resin infiltration methods may be used in combination or alone in the treatment of initial enamel lesions. Combining remineralizing agents with resin infiltration does not alter the efficacy of the treatment.

## Highlights

The use of remineralizing agents before resin infiltration does not alter the effectiveness of treatment for the start of demineralization located on the outer half of the enamel.

## Introduction

White spot lesions or early caries lesions are the first clinical manifestation of dental caries. Caries is defined as the subsurface porosity of enamel caused by demineralization and appears clinically as a milky white opacity on smooth surfaces. The outermost layer of enamel covering the lesion remains relatively intact and appears radiopaque on radiographs [1, 2]. Treatment of white spot lesions can be managed with conservative methods such as enhanced resin infiltration and some remineralizing agents [1, 3]. Using the Icon^®^ resin infiltration (DMG, USA), 15% HCl etchant can remove the surface layer of the decalcified area with a penetration depth of 58 ± 37 μm. This creates an opening in the lesion’s body, enabling the resin to block the pores. The lesion’s body is made watertight by applying resin with a refractive index (RI Icon = 1.44) similar to that of healthy enamel (RI = 1.63). This also functions by establishing a barrier within the lesion, thus preventing the diffusion of acids, rather than just on its surface [4]. The material’s superiority lies in its ability to penetrate the porosity of lesions due to its low viscosity [3, 5]. Resin infiltration offers numerous benefits, including preservation of tooth structure, enhanced stability in white spot lesions, prevention of caries progression, clogging of micropore forms in the lesion body, delay in the need for restoration, reduction in recurrent caries, and absence of inflammation. It also enhances esthetics through minimization of pulp and postoperative sensitivity, reduces the probability of periodontitis and gingivitis, and masks demineralized enamel [3]. Furthermore, besides resin infiltration, there are various other treatment alternatives available for treating initial enamel lesions. These include the remineralization process that uses different types of fluoridated and non-fluoridated remineralization agents [6, 7]. Remin Pro (VOCO, Germany) is a water-based remineralizing agent. It contains hydroxyapatite in the form of calcium and phosphate, as well as fluoride and xylitol. Hydroxyapatite is used to fill eroded enamel, while fluoride seals dentinal tubules, and xylitol functions as an antibacterial agent. Remin Pro has a high fluoride content of 1450 ppm that supports enamel remineralization [8]. It is assumed that this product is suitable for the management of dentinal hypersensitivity, prevention of enamel demineralization and support of remineralization of sub-enamel lesions [9, 10]. Additionally, Tooth Mousse (GC, Japan) contains casein phosphopeptide-amorphous calcium phosphate (CPP-ACP) technology. There is substantial evidence supporting the effectiveness of CPP-ACP in remineralizing initial caries and halting their advancement [11–13]. CPP-ACP efficacy has also been demonstrated in phosphopeptide-amorphouscariogenic bacteria increased colonization of commensal microorganisms and decreased dentin hypersensitivity [11, 14]. The mechanism of action of Mineral Gel (R.O.C.S., Germany) relies on releasing bioavailable calcium, phosphate, and magnesium, which can effectively treat early caries and erosive lesions. This product has also been proven in an in-vitro study to be effective in remineralizing white spots, reducing hypersensitivity, and improving the appearance of teeth [15]. Considering the importance of early diagnosis and treatment of white spot lesions, OCT (Optical Coherence Tomography) and Ultrasound evaluations have been used in many studies in the literature. There are also studies examining surface roughness, microhardness, and fluorescence properties [16–20]. Several studies have investigated the properties of resin infiltration or remineralization agents. In the clinic, resin infiltration and remineralizing agents, or a combination of both, are considered for the treatment of initial enamel lesions. However, few studies have evaluated the efficacy of the combination of these materials.

This study aimed to evaluate the efficacy of these agents using single and combined resin infiltration. Baseline, post-demineralization, and after treatment fluorescence (FluoreCam Daraza Therametric Technologies, USA; and DIAGNOdent Pen, Kavo, Germany), surface microhardness (HMV-2T, Shimadzu, Japan), Optical Coherence Tomography (3D OCT-1 Maestro, Topcon, Japan), Ultrasound (Novascope 4500, NDT, USA) and surface roughness (M300C, Mahr, Germany) data were obtained for all groups.

The hypotheses of this study were as follows:

1. The application of a remineralizing agent before resin infiltration results in increased fluorescence and OCT values which indicate lesion depth compared to their application alone.
2. The application of a remineralizing agent before resin infiltration results in increased surface properties including microhardness and surface roughness.

## Material and method

### Sample preparation

This study was approved by the Ethics Committee of Marmara University (reference number: 2022/94) and conducted in accordance with the Declaration of Helsinki. Informed consent was obtained from each patient for using extracted teeth. Eighty buccal enamel blocks (*n* = 10) without caries lesions were chosen in the present study utilizing periodontal or orthodontic 80 human molar extractions within the last 6 months. The number of samples was evaluated using G*power version 3.1.9.7 (α = 0.05, 1-β = 0.95) and the sample size was determined as 10 samples for each group based on the previous study [21]. Teeth were stored in distilled water after cleaning with a fluoride-free paste. The enamel areas from the crown of the teeth were cut under water. Enamel samples were examined under a stereomicroscope (Leica MZ7.5, Germany) at x20 magnification. They were embedded in acrylic to expose the enamel surfaces. The teeth were smoothed and standardized by polishing with silicon carbide paper ranging from 600 to 1200 grit. Teeth that were cleansed with distilled water.

### Artificial demineralization

Eighty buccal enamel samples were immersed in the demineralizing solution (Table 1) for 96 h. The DIAGNOdent Pen (Kavo, Germany) was used to monitor the lesions formed on the enamel, and samples in the range of 14–20 were selected for the study. The manufacturer proposed a score range of 14–20 to indicate the start of demineralization on the outer half of the enamel.

**Table 1:** Demineralization and remineralization solutions, their contents, and pH [22]

Solutions	Contents	pH
Remineralization	1.5 mM Ca(NO_3_)_2_∙4H_2_O, 0.9 mM NaH_2_PO_4_∙2H_2_O, 150 mM KCl, 0.1 mol/L Tris buffer, and 0.03 ppm F.	7
Demineralization	2.0 mM Ca(NO_3_)_2_∙4H_2_O, 2.0 mM NaH_2_PO_4_∙2H_2_O, 0.075 mM acetate buffer, and 0.02 ppm F.	4.7

*Abbreviations Ca(NO*_*3*_*)*_*2*_.*H*_*2*_*O*, calcium nitrate, *NaH*_*2*_*PO*_*4*_.*H*_*2*_*O* disodium hydrogen phosphate monohydrate, *KCl* potassium chloride, *F*, fluoride

### pH cycle and treatment procedures

**Fig. 1:**
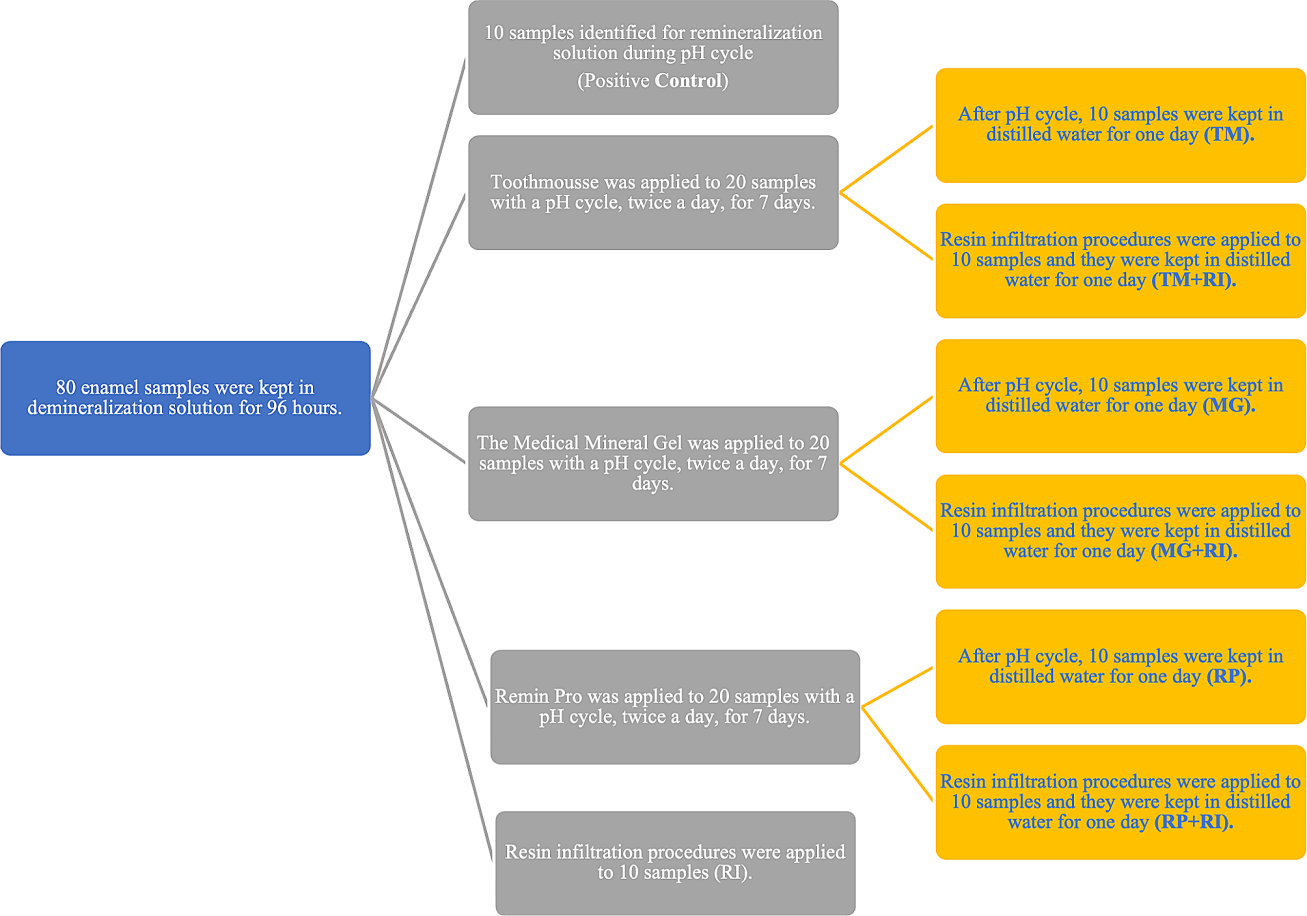
The treatment procedures and groups used in the study. RI: Only resin infiltration; TM: Only Tooth Mousse; TM+RI: Resin Infiltration after Tooth Mousse; MG: Only Medical Mineral Gel; MG+RI: Resin Infiltration after Medical Mineral Gel; RP: Only Remin Pro; RP+RI: Resin Infiltration after Remin Pro; Positive Control: Samples immersed in remineralization solution

The demineralized enamel samples were separated randomly into eight groups (*n* = 10). The groups and agents were as follows: Positive Control (remineralization solution); Tooth Mousse (GC, Japan)/TM; Remin Pro (VOCO, Germany)/RP; Medical Mineral Gel (R.O.C.S., Germany)/MG; ICON Resin Infiltration (DMG, Germany)/RI; Tooth Mousse before ICON resin infiltration/TM + RI; Remin Pro before ICON resin infiltration/RP + RI; Medical Mineral Gel before ICON resin infiltration/MG + RI (Fig. 1).

**Fig. 2:**
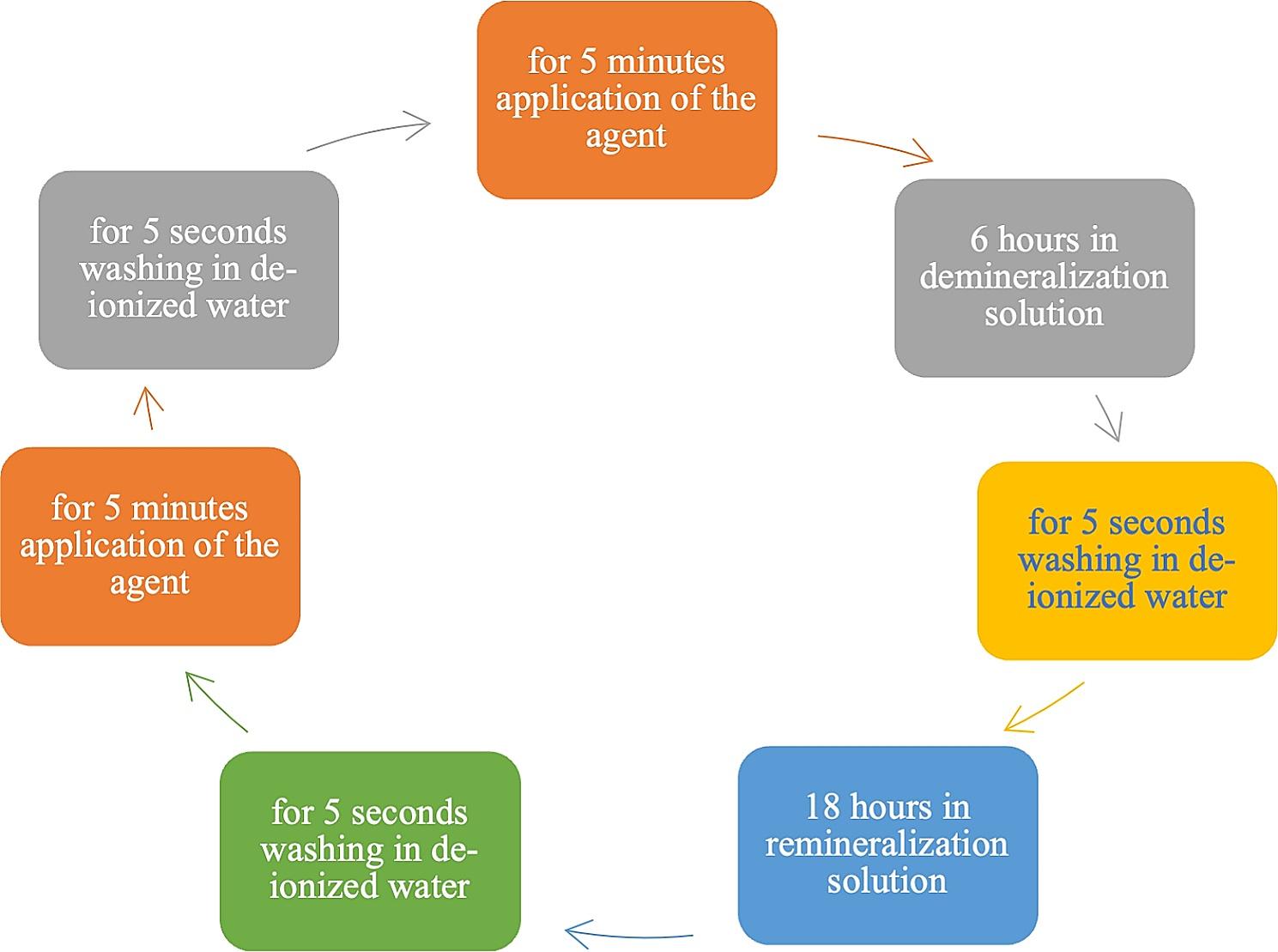
Application of remineralizing agents and demineralization/remineralization cycle

The one group only utilizing ICON resin infiltration (RI) was not included in the pH cycle. The positive control group was subjected to a remineralization solution without the application of any remineralizing agent or resin infiltration. For the other six groups, the remineralization agent was applied twice daily for seven days in a specific regimen. This regimen consisted of a five min. application of the agent, followed by 6 h in demineralization solution, and then a 5 s. rinsing with de-ionized water. This was followed by 18 h in a remineralization solution [22] (Table 1), another 5 s. rinsing with de-ionized water, and a final five min. application of the agent followed by a 5 s. rinse with de-ionized water (Fig. 2). Three agents were used for remineralization during pH cycling: Tooth Mousse (TM), Medical Mineral Gel (MG), and Remin Pro (RP). In the resin infiltration included groups (TM + RI, RP + RI, and MG + RI), after a pH cycle, ICON-Etch was applied for 2 min., followed by a 30 s. rinse and a 30 s. ICON-Dry application. The Icon-Infiltrant was then left for 3 min. and polymerized for 40 s. Afterward, the ICON-Infiltrant was reapplied for 1 min. then polymerized again for 40 s. (Table 2). The finishing and polishing procedure of resin infiltrant was performed using 4-step grinding discs (Finishing Discs, Bisco, USA).

**Table 2:** Remineralization agents, resin infiltration, their contents, and application procedures are recommended by the manufacturer

Material	Contents	Manufacturers’ recommendation*
ICON Resin Infiltration (DMG, Germany)	TEGDMA based resin matrix, initiator – additives	1. Apply Icon-Acid (15% HCl), rinse with water for 2 min; wait 30 s and air dry.
		2. Apply Icon-Dry for 30 s.
		3. Icon Infiltrant, wait for 3 min, polymerization for 40 s.
		4. Icon Infiltrant, wait for 1 min, polymerization for 40 s.
Tooth Mousse (GC Corp., Japan)	10% Casein Phosphopeptide Amorphous Calcium Phosphate (CPP-ACP)	1. After brushing your teeth, squeeze a small amount of GC Tooth Mousse onto a clean finger.
		2. Apply to all teeth with a clean finger and use your tongue to spread around evenly.
		3. Leave GC Tooth Mousse on teeth for a minimum of 3 min avoiding spitting out and swallowing. For better results, allow GC Tooth Mousse/Plus to remain in contact with your saliva for an additional 1–2 min.
		4. Spit out the excess. Any GC Tooth Mousse remaining on the surface can be left to gradually dissipate. Avoid rinsing, and do not eat or drink for 30 min following application.
Medical Mineral Gel (R.O.C.S., Germany)	Calcium glycerophosphate (C_3_H_7_CaO_6_P), Magnesium Chloride (MgCl_2_) and 10% Xylitol	1. Thoroughly clean your teeth with a toothbrush prior to applying the gel.
		2. Apply the gel onto the toothbrush and distribute evenly over the dentitions. Do not rinse the mouth and refrain from food or drink for the next 40–50 min. It is recommended to apply the gel twice a day: after breakfast and before bed.
		3. Use daily for 2 weeks, 2–3 times per year.
Remin Pro (VOCO GmbH, Germany)	Sodium Fluoride (1450 PPM F^−^), Hydroxylapatite and Xylitol	1. Apply a pea-size amount of Remin Pro to the teeth with a finger or suitable instrument (toothbrush or swab) and distribute.
		2. Distribute the remaining Remin Pro in your mouth with your tongue. Remin Pro and the saliva should remain in your mouth for as long as possible (minimum of 3 min) for an optimal result.
		3. Spit out the remaining amount. Rinsing should be avoided if possible. Wait at least 30 min after the treatment before eating or drinking.

*Abbreviations TEGDMA* triethyleneglycol dimethacrylate, *CPP-ACP* Casein Phosphopeptide Amorphous Calcium Phosphate, *C*_*3*_*H*_*7*_*CaO*_*6*_*P* Calcium glycerophosphate, *MgCl*^*2*^ Magnesium Chloride, *PPM* Parts Per Million

*The protocol for this in vitro study is shown in Fig. 2

### Measurements

All samples were measured at baseline, after demineralization, and after treatment procedures with these methods:

### Assessment of surface properties

It was measured at three different points on an HMV-2T (Shimadzu, Japan) microhardness device with a Vickers elongated diamond tip and an x40 magnification lens a 100-gram load was applied to the surface for 10 s., with three separate indentations taken, and the mean value considered. A precision microscope with microhardness evaluation has been one of the most preferred methods in-vitro studies to assess remineralization x400 magnification was used to measure the indentations and the diagonal length of the indentation was measured with the built-in scale microscope, and Vicker’s Hardness Numbers were converted to surface microhardness values (HV).

The enamel surface roughness parameter (Ra) was measured by tracing a line and calculating the arithmetic mean of the absolute deviation values of the roughness profile from the mean line. Roughness measurements were made using the M300C (Mahr, Germany) contact profilometer.

One sample of each group was sectioned longitudinally in a buccolingual direction with a diamond saw (Isomet 1000, Buehler, USA) under water. The buccal and interface (treatment/tooth) surfaces of the unpolished samples were analyzed by Scanning Electron Microscopy/Energy Dispersive X-ray Spectrometry (SEM/EDX) (SU3500, Hitachi, Japan) with an acceleration voltage of 10 kV and magnification of x1000. The examination focused on the mid-section of the sample’s surface and interface, with particular attention to the presence of O, P, Ca, N, F, Na, C, Sr, Sn, Si, Mg, Al, Zn, and Sb elements. The elemental atomic percentages were recorded. Before scanning and analyzing, the samples were coated with a thin layer of gold.

### Assessment of fluorescence properties

The DIAGNOdent Pen was calibrated before each measurement, and a conical tip was used to make a right-angle direct application on clean tooth surfaces. Three measurements were taken from each sample and values (DDP) were recorded.

Fluorescence differences within the lesion image were measured for size and intensity using FluoreCam (Daraza Therametric Technologies, USA). The FluoreCam software analyzed the measurements, with size referring to the area of the lesion and intensity indicating the degree of mineral loss. The alteration was verified through the “Compare” within the FluoreCam system.

### Assessment of lesion depth

The SD-OCT system (Maestro-2, Topcon Healthcare, USA) used in this study has a broadband diode at 840 nm as a low coherent light source and a frequency rate of 50 Hz. The scanning probe, which was handheld and linked to the OCT system, was positioned 6 cm away from the samples’ surface. The scanning beam was directed at an angle of approximately 90° to the surface. OCT raw data were analyzed using a custom code for image analysis in the ImageJ software program (ImageJ version 1.45 S, National Institutes of Health, USA). The software’s threshold function allows for the identification of acceptable intensity values that correspond to the visible edge of enamel lesions. This indicates the demineralization front or optical lesion depth (OLD). The OLD was calculated over a specified region of interest (ROI, width 200 μm × optical depth 400 μm).

After the enamel samples were coated with ZG-F Ultrasonic Coupling Gel (GE Inspection Technologies, Germany), a probe was utilized to perform measurements on the enamel surfaces. Novascope 4500 ultrasound device (NDT, USA) was used to measure from three distinct points on each surface, and the mean value was recorded (ULS).

### Statistical analysis

The data were analyzed in the R program with the WRS2 package. The Shapiro-Wilk Test examined the conformity to the normal distribution. Data that are not normally distributed according to group and pH cycle were analyzed with the Two-Way Robust ANOVA Test and multiple comparisons were made with the Bonferroni Test. ICC (Intra-Class Correlation Coefficient) was used to examine the agreement between the measurements. The significance level was set at *p* < 0.050.

## Results

### Assessment of surface properties

There was no statistically significant difference in HV between the groups (*p* = 0.582). However, the effect of the treatment procedure on HV was significant for all groups (*p* < 0.001). The interaction of the group and procedure was statistically significant on the mean values of the HV (*p* = 0.047). The lowest HV value was obtained at 152.3 in TM after the remineralization procedure (Table 3).

**Table 3:** Descriptive statistics and comparison of the surface microhardness values

Group	Surface Vicker’s Microhardness (HV)			
	Baseline	After demineralization	After treatment	Group Main Effect
TM	330.83 (162.33–506)^ABC^	200.83 (137–290)^AB^	152.3 (85,67–266)^A^	193 (85.67–506)
RP	302.5 (267.67-389.33)^BC^	250.17 (130.33-296.67)^ABC^	165.5 (97.73-244.67)^ABC^	250.17 (97.73-389.33)
MG	285.17 (229.67-349.67)^BC^	262.67 (131.67–312)^ABC^	236.17 (100.77-336.67)^ABC^	257.5 (100.77-349.67)
+Control	286 (219-427.33)^ABC^	252 (141.33-323.33)^ABC^	216 (183-257.67)^ABC^	253.33 (141.33-427.33)
RI	414.67 (177.33-733.33)^ABC^	196.33 (150–340)^ABC^	261.67 (106–407)^ABC^	261.67 (106-733.33)
TM + RI	273.67 (227.67–499)^BC^	249.33 (183-296.67)^ABC^	286 (161-362.33)^ABC^	266 (161–499)
RP + RI	290 (171.67-484.33)^C^	252.33 (137.33-324.67)^ABC^	190.33 (156-282.33)^ABC^	252.33 (137.33-484.33)
MG + RI	266.33 (211.33-355.67)^BC^	207.5 (169-281.67)^ABC^	215.83 (141-452.67)^ABC^	227 (141-452.67)
**Procedure Main Effect**	288.67 (162.33–733,33)^b^	236.83 (130.33–340)^a^	210 (85.67–452.67)^a^	250.5 (85.67-733.33)
		**Q**	***p***	
	**Group**	0.807	0.582	
	**Procedure**	10.626	**< 0.001**	
	**Group x procedure**	23.889	**0.047**	

Median (minimum–maximum), Q: Two-way Robust ANOVA Test Statistics, a-c and A-C: The same letter is not significantly different

No statistical difference in Ra between groups (*p* = 0.963). Likewise, the main effect of the treatment procedure was not significant on Ra (*p* = 0.984). The lowest Ra was obtained in the TM + RI group after remineralization (Table 4).

**Table 4:** Descriptive statistics and comparison of the values of surface roughness

Group	Surface Roughness (Ra)			
	Baseline	After demineralization	After treatment	Group Main Effect
TM	0.38 (0.18–0.95)	0.72 (0.39–1.59)	0.96 (0.46–1.48)	0.72 (0.18–1.59)
RP	0.31 (0,2 − 0,75)	0.94 (0.54–1.17)	0.86 (0.55–1.39)	0.74 (0.2–1.39)
MG	0.42 (0.24–0.57)	0.63 (0.46–1.71)	0.71 (0.39–0.92)	0.56 (0.24–1.71)
+Control	0.51 (0.2–0.93)	0.85 (0.48–1.24)	0.95 (0.35–1.91)	0.68 (0.2–1.91)
RI	0.47 (0.24–1.3)	0.68 (0.43–2.44)	0.82 (0.17–1.8)	0.68 (0.17–1.3)
TM + RI	0.31 (0.17–0.78)	0.99 (0.43–2.4)	0.65 (0.29–0.99)	0.65 (0.17–2.4)
RP + RI	0.38 (0.22–0.9)	0.82 (0.26–1.28)	0.94 (0.45–1.39)	0.64 (0.22–1.39)
MG + RI	0.57 (0.21–1.11)	0.98 (0.35–2.54)	0.87 (0.59–1.11)	0.74 (0.21–1.11)
**Procedure Main Effect**	0.43 (0.17–1.3)	0.85 (0.26–2.54)	0.82 (0.17–1.91)	0.68 (0.17–1.3)
		**Q**	***p***	
	**Group**	0.277	0.963	
	**Procedure**	0.016	0.984	
	**Group x procedure**	18.273	0.195	

Median (minimum–maximum), Q: Two-way Robust ANOVA Test Statistics

**Fig. 3:**
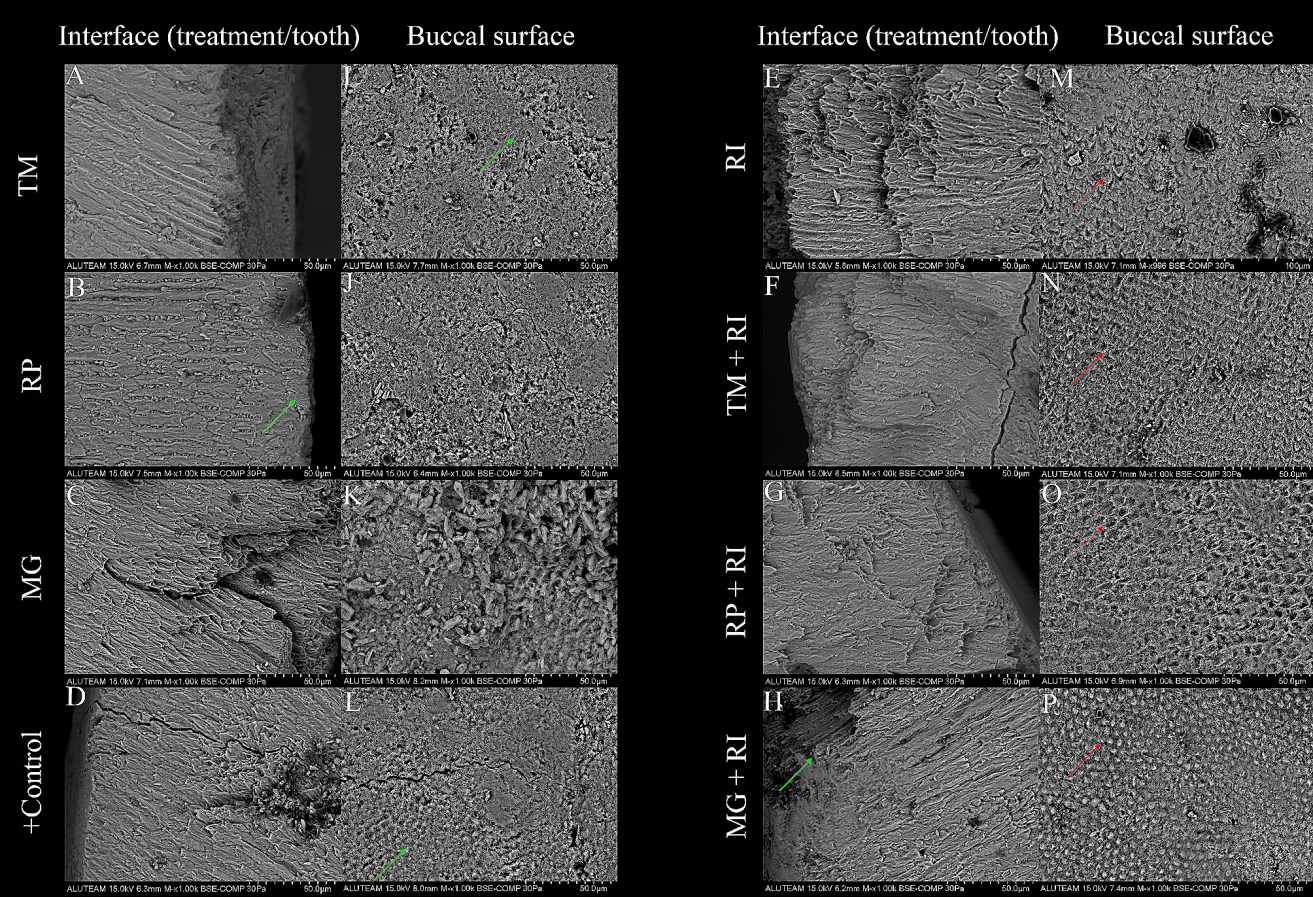
SEM images after treatment at x1000 magnification. RI: Only resin infiltration; TM: Only Tooth Mousse; TM+RI: Resin Infiltration after Tooth Mousse; MG: Only Medical Mineral Gel; MG+RI: Resin Infiltration after Medical Mineral Gel; RP: Only Remin Pro; RP+RI: Resin Infiltration after Remin Pro; Positive Control: Samples immersed in remineralization solution with pH cycle. A-H: interface (treatment/tooth) images when samples were sectioned longitudinally in a buccolingual direction, I-P: buccal surface images. The occluded prisms (green arrow) and fish-scale-like enamel appearance (red arrow) were observed

It was observed that all treatment procedures produced results comparable to the positive control group in the SEM images taken at the interface after treatment. Interface SEM images of the treatment groups showed similarity to the positive control group. Surface images revealed similar surface roughness and homogeneity among the remineralization treatment groups. The appearance of occluded microporosities, comparable to that of the positive control group, is observed in the buccal and interface (treatment/enamel) surface images (green arrow). However, in the resin infiltrated groups, applying HCl and not polishing resulted in a fish-scale-like enamel appearance (red arrow) (Fig. 3). The lowest and highest Ca ratio (%atomic) was observed in the interface of TM + RI and interface of TM, respectively. The RP surface showed the highest amount of F ratio (Table 5).

**Table 5:** Elemental composition (atomic%) for each treatment group using EDS analysis

Elements	Surface								Interface							
	TM	RP	MG	Control	RI	TM + RI	RP + RI	MG + RI	TM	RP	MG	Control	RI	TM + RI	RP + RI	MG + RI
O	63.08	65.06	62.72	64.96	65.45	64.14	64.12	65.37	62.06	62.58	63.13	64.98	64.56	65.81	64.64	64.71
P	10.38	7.45	7.83	7.79	6.27	8.63	8.79	6.23	10.81	10.92	9.83	8.89	7.36	5.31	7.36	6.54
Ca	15.07	12.04	17.08	11.38	9.35	13.6	12.94	9.27	17.83	16.8	15.91	10.17	12.61	8.46	13.07	10.7
N	0	10.83	4.23	7.64	7.99	6.04	5.35	7.07	0	1.5	3.82	6.16	7.81	9.04	9.53	7.25
F	0	0.56	0	0.23	0.29	0.2	0.38	0.24	0	0.16	0.07	0.14	0.09	0.2	0.29	0.28
Na	0.37	0.15	0.15	0.21	0.19	0.22	0.25	0.21	0.73	0.42	0.47	0.36	0.32	0.29	0.25	0.26
C	10.51	2.52	6.62	6.24	9.29	5.84	6.83	10.54	8.02	6.1	5.37	9.98	5.98	10.03	3.78	8.71
Sr	0.06	0.03	0	0.04	0.03	0.04	0.05	0.03	0.05	0.04	0.05	0.03	0.06	0.03	0.04	0
Sn	0.03	0.13	0.1	0.1	0.09	0.16	0.14	0.09	0	0.15	0.13	0.09	0.12	0.07	0.11	0.1
Si	0.21	0.28	0.19	0.3	0.1	0.14	0.13	0.17	0.2	0.12	0.11	0.16	0.09	0.09	0.08	0.19
Mg	0.12	0.03	0.04	0.08	0.06	0.07	0.07	0.08	0.15	0.14	0.16	0.1	0.1	0.1	0.07	0.11
Al	0.05	0.1	0.09	0.06	0.25	0.04	0.11	0.12	0.09	0.13	0.03	0.06	0	0.02	0.02	0.06
Zn	0.12	0.03	0.05	0.09	0.06	0.06	0.08	0.02	0.06	0	0	0.02	0.02	0.06	0.04	0.03
Sb	0	0.73	0.87	0.71	0.57	0.81	0.78	0.55	0	0.94	0.92	0.6	0.74	0.49	0.74	0.61

*Abbreviations, O* oxygen, *P* phosphor, *Ca* calcium, *N* nitrogen, *F* fluoride, *Na* sodium, *C* carbon, *Sr* strontium, *Sn* tin, *Si* silicon, *Mg* magnesium, *Al* aluminum, *Zn* zinc, *Sb* antimony

### Assessment of fluorescence properties

There was no statistically significant difference in DDP between the groups (*p* = 0.923), but the treatment procedures had a significant effect for all groups (*p* < 0.001). The lowest DDP value was found in the RP + RI group after the treatment (Table 6).

FluoreCam data has been evaluated for both size and intensity. FluoreCam size and intensity values were not statistically different between groups (*p* = 0.269; *p* = 0.793, respectively). On the contrary, treatment procedures yielded a statistically significant impact on both the size and intensity (*p* < 0.001) (Table 6).

**Table 6:** Descriptive statistics and comparison of DIAGNOdent Pen and FluoreCam values

Group	DIAGNOdent Pen (DDP)				FluoreCam (FCam)							
					Size				Intensity			
	Baseline	After demineralization	After treatment	Group Main Effect	Baseline	After demineralization	After treatment	Group Main Effect	Baseline	After demineralization	After treatment	Group Main Effect
TM	4 (2.67–5.33)	8.17 (6.67–12.33)	4.17 (2.67-7)	4.83 (2.67–12.33)	3.45 (1.03–6.21)	4.44 (1.06–12.24)	2.3 (0.67–6.33)	3.56 (0.67–12.24)	-11.67 (-15.01- -5.94)	-12.61 (-22.47- -8.09)	-7.69 (-19.12- -5.22)	-10.8 (-22.47- -5.22)
RP	5.33 (3.33-7)	7.83 (6.33–12.67)	4.67 (3.33–6.33)	5.67 (3.33–12.67)	4.22 (1.27–7.6)	5.23 (3.88–8.89)	4.31 (1.96–5.44)	4.51 (1.27–8.89)	-11.7 (-15.99- -7.69)	-13.24 (-17.59- -8.79)	-9.5 (-16.61- -7.37)	-11.61 (-17.59- -7.37)
MG	5.5 (3-6.33)	8 (5-11.33)	3.83 (3-6.33)	5.67 (3-11.33)	2.75 (1.09–4.64)	4.06 (1.03–7.83)	1.89 (0.39–7.59)	2.71 (0.39–7.83)	-12.12 (-24.05- -3.99)	-13.62 (-19.78- -7.99)	-7.89 (-18.17- -3.86)	-10.3 (-24.05- -3.86)
+Control	3.83 (3.33-6)	7.17 (6.33-13)	6 (4-14.67)	6.17 (3.33–14.67)	5.19 (1.73–8.31)	6.28 (2.71–11.12)	2.57 (0.74–4.8)	4.31 (0.74–11.12)	-13.33 (-21.24- -7.65)	-13.29 (-21.06- -9.03)	-8.19 (-12.45- -5.17)	-10.79 (-21.24- -5.17)
RI	4.33 (3.33–6.67)	8 (6-13.67)	3.67 (2.67–6.67)	5.33 (2.67–13.67)	4.71 (0.68–8.3)	4.09 (1.73–6.5)	2.31 (0.6–3.35)	3.35 (0.6–8.3)	-12.22 (-20.03- -6.48)	-10.74 (-15.33- -7.12)	-7.34 (-13.74- -4.96)	-10.46 (-20.03- -4.96)
TM + RI	4.33 (4-6.33)	8 (6.67–13.33)	5 (3.33–7.67)	5.67 (3.33–13.33)	3.1 (1.39–8.65)	5.24 (2.98–9.85)	2.63 (0.89–7.33)	3.79 (0.89–9.85)	-11.99 (-15.79- -6.12)	-11.66 (-15.93- -8.06)	-7.85 (-13.64- -5.27)	-10.37 (-15.93-6.12)
RP + RI	4.67 (3.67–6.67)	8 (6.33–16.67)	2.33 [17]	5 (1-16.67)	2.34 (0.93–4.73)	4.73 (1.95–8.46)	1.98 (0.65–3.32)	2.64 (0.65–8.46)	-11.29 (-14.44- -5.88)	-10.86 (-15.35- -8.14)	-7.06 (-13.17- -5.3)	-10.11 (-15.35- -5.3)
MG + RI	4.5 [3-8]	8.17 (6-12.33)	5 (3.67-6)	5.33 (3-12.33)	5.26 (1.41–7.13)	5.27 (1.45–9.49)	2.43 (1.05–4.74)	4.15 (1.05–9.49)	-13.9 (-16.79- -7.11)	-10.98 (-29.59-14.3)	-9.81 (-16.31- -8.65)	-11.25 (-29.59-14.3)
**Procedure Main Effect**	4.67 (2.67-8)^a^	8 (5-16.67)^b^	4.67 (1-14.67)^a^	5.33 (1-16.67)	3.69 (0.68–8.65)^c^	5.02 (1.03–12.24)^b^	2.59 (0.39–7.59)^a^	3.62 (0.39–12.24)	-12.21 (-24.05-6.12)^b^	-11.57 (-29.59-14.3)^b^	-8.53 (-19.12 -3.86)^a^	-10.83 (-29.59-14.3)
			**Q**	***p***			**Q**	***p***			**Q**	***p***
	**Group**		0.365	0.923		**Group**	1.257	0.268		**Group**	0.554	0.793
	**Procedure**		27.253	**< 0.001**		**Procedure**	9.61	**< 0.001**		**Procedure**	10.021	**< 0.001**
	**Group x procedure**		21.214	0.096		**Group x procedure**	10.273	0.742		**Group x procedure**	3.692	0.997

Median (minimum – maximum), Q: Two-way Robust ANOVA Test Statistics, a-c: The same letter is not significantly different

### Assessment of lesion depth

**Fig. 4:**
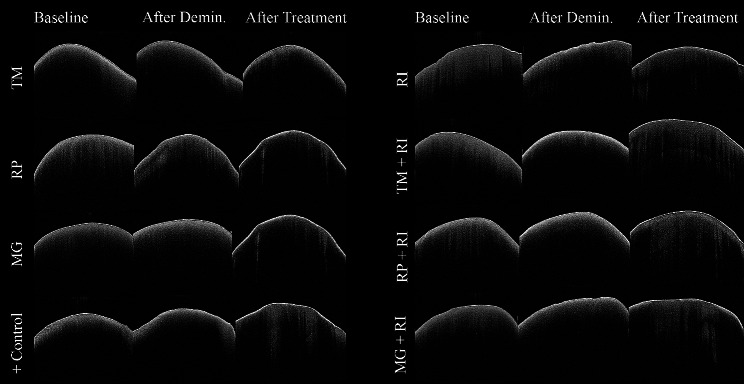
SD-OCT images of representative enamel surface at baseline, after demineralization, and after treatments

The treatment procedures effect was statistically significant on OLD median values (*p* < 0.001). During the pH cycling period, the samples treated with all the remineralization agents showed remineralization that reduced the OLD, however, there were no statistically significant differences between the groups (*p* = 0.884). The highest median value was recorded in TM + RI after the pH cycling (102.5) (Table 7). B-scan SD-OCT images of representative enamel surfaces at baseline, after demineralization, and after treatments are shown in Fig. 4.

**Table 7:** Descriptive statistics and comparison of OCT and ultrasound values

Group	Optical Lesion Depth (OLD)				Ultrasound (ULS)			
	Baseline (µm)	After demineralization (µm)	After treatment (µm)	Group Main Effect	Baseline	After demineralization	After treatment	Group Main Effect
TM	106 (80–140)	162.51 (102.01–224)	80.05 (71.03-145.06)	106.5 (71.03–224)	1.38 (1.24–1.86)^ABC^	1.09 (0.93–1.31)^AB^	2.38 (2.3–2.51)^D^	1.38 (0.93–2.51)
RP	111.26 (79.01-195.01)	141.51 (88.67-228.01)	69.93 (54-165.05)	111.26 (54-228.01)	1.39 (0.87–2.08)^ABC^	1.08 (0.8–4.08)^AB^	2.34 (2.28–2.4)^D^	1.51 (0.8–4.08)
MG	101.04 (88.01–138)	152 (105.5-181.01)	85.08 (55-148.5)	116 (55-181.01)	1.31 (1.06–3.9)^AC^	1.04 (0.86–1.36)^AB^	2.39 (2.32–2.43)^D^	1.35 (0.86–3.9)
+Control	112 (92.51-169.05)	147 (102.02-199.01)	101.01 (84.01-182.06)	130.05 (84.01-199.01)	1.35 (1.18–2.03)^ABC^	1.12 (0.63–1.36)^AB^	2.33 (2.24–2.43)^D^	1.39 (0.63–2.43)
RI	91.5 (65.03-188.01)	125 (104.33-247.01)	91.01 (61–122)	105.01 (61-247.01)	1.64 (1.31–4.29)^C^	1.17 (0.86–4.28)^AB^	2.36 (2.29–2.42)^D^	1.73 (0.86–4.29)
TM + RI	104 (83–155)	141.01 (112.51-214.01)	102.5 (82–143)	111.01 (82-214.01)	1.16 (0.97–1.71)^AB^	1.09 (0.87–1.27)^AB^	2.34 (2.3–2.39)^D^	1.27 (0.87–2.39)
RP + RI	97.01 (74.01-176.01)	126 (100.01-229.06)	87.04 (67.67–122)	100.01 (67.67-229.06)	1.3 (1.18–1.62)^AC^	1.1 (0.94–1.31)^AB^	2.67 (2.33–2.92)^D^	1.31 (0.94–2.92)
MG + RI	120.5 (77.01-259.03)	146.01 (92–296)	82 (60.34–159)	123.52 (60.34–296)	1.26 (0.99-2)^ABC^	1.04 (0.88–1.37)^B^	2.38 (2.32–2.54)^D^	1.35 (0.88–2.54)
**Procedure Main Effect**	104.01 (65.03-259.03)^c^	144.78 (88.67–296)^b^	90.5 (54-182.06)^a^	109.54 (54–296)	1.34 (0.87–4.29)^c^	1.1 (0.63–4.28)^b^	2.36 (2.24–2.92)^a^	1.37 (0.63–4.29)
		**Q**	***p***			**Q**	***p***	
	**Group**	0,430	0.884		**Group**	2.330	0.051	
	**Procedure**	14,920	**< 0.001**		**Procedure**	179.900	**< 0.001**	
	**Group x procedure**	14,418	0.419		**Group x procedure**	476.930	**< 0.001**	

Median (minimum – maximum), Q: Two-way Robust ANOVA Test Statistics; a-c and A-D: The same letter is not significantly different

There was no statistically significant difference in ULS between the groups (*p* = 0.051) but at the same time treatment procedure effect and group and procedure interaction were statistically significant on ULS for all groups (*p* < 0.001). The highest ULS was obtained after treatment in RP + RI, with 2.67 (Table 7).

## Discussion

Initial demineralized enamel lesions might be remineralized with appropriate treatment if the outer surface of the enamel is intact [8, 21]. Recently, resin infiltration has become one of the treatment options to cure and arrest enamel caries and it is an alternative treatment method to arrest carious lesions and enhance the surface properties of the teeth [2]. Therefore, the present study was designed to evaluate the effects of various remineralization agents and their combination with resin infiltration. Decreasing the enamel lesion before resin infiltration reduces the area to be infiltrated, potentially leading to higher success rates. The study selected remineralizing agents based on their effectiveness in previous clinical studies. Moreover, many studies are comparing these agents with each other [15, 23–26]. Yavuz et al. compared three different remineralization agents which contain CPP-ACP, CPP-ACP with fluoride, and calcium glycerophosphate, and found all remineralization agents were effective in increasing HV values, however, there was no statistical difference between the treatment groups [16]. Vyavhare et al. stated MI Paste should not be used as an alternative to fluoride, but it can be used as an adjunct agent to fluoride because of its lower remineralization potential [27]. On the contrary, Leila et al. found MI Paste more efficient than Remin Pro as a remineralization agent [28].

The efficacy of remineralizing agents and resin infiltration can be determined using methods such as microhardness evaluation, optical coherence tomography, and fluorescence methods [1, 2, 16, 18, 26]. One of these methods is the DDP which is based on laser fluorescence. Previous studies reported DDP as a reliable and quantitative method to detect or monitor lesion progression on smooth enamel surfaces [17, 29]. It has been reported that remineralization can be evaluated by laser fluorescence measurement using DDP. The DDP scores decreased post-resin infiltration and post-remineralization application [29, 30]. However, resin infiltration was more successful than fluoride varnish in the treatment of initial enamel lesions [31]. Some previous studies found a significant correlation between DDP and HV values after the remineralization process [32]. A decrease in DDP values was observed in all groups after the treatment procedures in this study. The lowest DDP value was found in the “Remin Pro before resin infiltration group” after the treatment. Although, there was no statistical difference found between the treatment groups, HV and DDP showed similar results in the present study. HV evaluation has been one of the most preferred methods in-vitro studies to assess remineralization [33]. It is known that HV values increase as the mineral content of the tooth tissue increases after remineralization The remineralization potential of an agent is associated by its ability to increase HV as near as intact enamel. A recently released meta-analysis showed CPP-ACP can remineralize initial enamel lesions and increase the HV values [13], on the contrary, Pintanon et al. reported that applying CPP-ACP-containing paste daily did not increase the HV of the enamel lesion [34]. In the present study, all mean microhardness values decreased after the demineralization process. After the remineralization process, the lowest values were obtained from Tooth Mousse group, and the highest values were recorded in the Resin Infiltration after Tooth Mousse group however no statistical differences were found between all groups [35, 36].

In different studies, laser fluorescence methods were used to evaluate the effectiveness of demineralization and remineralization. FluoreCam is another fluorescence method used in the study to observe the effect of remineralizing agents, which uses light fluorescence with an extraoral apparatus to detect and follow up demineralized tooth tissue [30, 37]. Gungor et al., evaluated the remineralization efficiency of the agents using FluoreCam. They stated that fluoride varnish was the most effective agent [30]. Bilgin et al. applied Sodium fluoride (NaF) dentifrice, Tooth Mousse, Remin Pro, Pro fluoride three times a day for 1 min for 21 days. Researchers evaluated the remineralization efficiency of the agents using FluoreCam. They reported that all treatment groups showed remineralization according to FluoreCam size values, which represent lesion size, and intensity values, which indicate the mineral content of the lesion [38]. According to the results of the present study, the size values of demineralized areas in all groups decreased, and the intensity values increased after the remineralization process with no statistical difference among the groups. The treatment procedures yielded a statistically significant impact on both the size and intensity of FluoreCam. Earlier research has demonstrated that OCT can evaluate early enamel lesions and monitor the remineralization process or resin infiltration treatment [16, 23, 39]. A study claimed that the pH cycling regimen led to a substantial reduction in the OLD as the backscattering decreased. However, the efficacy of the evaluated remineralizing agents (Tooth Mousse, MI Paste Plus, Remin Pro, and Medical Mineral Gel) seems to be comparable to one another [16]. In this in-vitro study, as in previous ones, all processing methods and pH cycles had a significant effect on OLD but did not differ between groups. The lack of significant difference between the different treatments may be due to the fact that this study used the start of demineralization on the outer half of the enamel.

It is necessary to obtain quantitative and standard data in order to carry out controlled in-vitro studies. Ultrasonic systems are convenient for determining the demineralization depth of teeth as they are quick and non-destructive techniques [17, 40]. Kim et al. determined the depth of white spot lesions with a high-frequency ultrasound imaging system and reported that their results were in good agreement with the depths obtained with micro-CT images. They stated that high-frequency ultrasound imaging may be useful for detecting white spot lesions and quantitatively determining the depth of demineralization because it is a non-destructive technique with no ionizing radiation [41]. This in-vitro study demonstrated that remineralization agents significantly impacted ULS. However, there was no difference between the resin infiltration, remineralizing agents, and remineralizing agents’ application before the infiltration of the resin. Among the methods evaluated, the pH cycle and treatment procedure were found to have a statistically significant effect on DDP, FluoreCam size and intensity, HV, OLD, and ULS. The highest ULS was obtained after the “Remin Pro before resin infiltration” group with 2.67 values. These results represented all treatment options with or without resin infiltration might be effective on remineralization so the first hypothesis that “the application of a remineralizing agent before resin infiltration results in increased fluorescence and OCT values which indicate lesion depth compared to their application alone” was rejected.

Concerning surface microhardness, a previous study reported that HV values after resin infiltration were higher than CPP-ACP treated group meanwhile the control group had the lowest HV values [42]. They also indicated that surface Ra values of the CPP-ACP treated group were higher than resin infiltrated group. They stated resin infiltration, as a minimally invasive method, protects the external intact enamel surface, which provides a smooth layer of the enamel. A previous studies pointed out that Remin Pro is an effective agent for remineralization of initial enamel caries [9, 43]. Remin Pro consists of hydroxyapatite, which can infuse in the porosities of the lesion, and fluoride, which strengthens the enamel structure [9]. Mielcazrek. et al. demonstrated that the toothpaste containing nanohydroxyapatite increased HV and decreased Ra of initial enamel lesions [27]. If demineralized enamel microporosities are remineralized or infiltrated successfully Ra of the treated samples is expected to decrease [44]. Although Ra values of the treatment groups in the present study were found to be higher than the baseline values recorded from healthy enamel samples. Additionally, the lowest Ra was obtained in the “Tooth Mousse before the resin infiltration” group after remineralization. Bakaa. et al. stated that the Ra decreased after resin infiltration [45]. On the other hand, Ulrich. et al. stated that the resin infiltration does not improve the Ra. They indicated that the microporosities after hydrochloric acid application were not filled with resin completely, consequently, the Ra increased [46]. According to the study of Katarzyna et al., remineralization agents and resin infiltration methods applied to the demineralized area can reduce the Ra [47]. In a study by Parastou et al., it was found that it increased the HV of demineralized enamel treated with a remineralization agent [48]. The results of the current study indicate that applying resin infiltration after remineralization agents did not have a significant statistical impact on HV, DDP, or Ra values. In addition, the resin infiltration method applied in addition to the use of remineralization agents was not statistically different. Based on this result, the second hypothesis that “the application of a remineralizing agent before resin infiltration results in increased surface properties including microhardness and surface roughness” was rejected. In the study of Aliaa et al., it was found that the enamels on which the resin infiltration method used in combination with remineralization agents were applied were more mechanically resistant than the demineralized enamel. Additionally, the resin infiltration method applied in addition to the use of remineralization agents was not statistically different [21].

In SEM images, the surface topography of the remineralizing agents was similar, and all groups provided occlusion like the positive control group in interfaces. Molaasadolah et al. found that the remineralization agents (Remin Pro, Tooth mousse, and Fluoride Gel) in their study demonstrated comparable porosities and surface coverage layer on the enamel prisms to both the control group and each other [26]. In some studies, an irregular, pitted honeycomb appearance was observed after demineralization or HCL acid etching because prism cores were preferentially dissolved. Likewise, in this study like previous studies, infiltrated resin showed a similar appearance because resin-infiltrated lesions were irregular with the exposure of enamel crystals under the resin matrix [3, 49]. The lack of difference between resin infiltration and combination with remineralizing agents might be due to the roughening effect of ICON-Etch (HCL). Still, microporosities were occluded and exhibited a largely intact and uniform surface [22]. In an in-vitro study, the SEM image showed a fish-scale appearance on the unpolished surface with resin infiltration [50], the surfaces remained unpolished in the resin-infiltrated groups and displayed the same image in the present study. Furthermore, it is worth noting that the image exhibited a fish-scale-like appearance in the RI, TM + RI, and MG + RI groups, whereas it displayed a honeycomb image in the RP + RI group. The levels of Ca and P, the main components of enamel, indicate demineralization. F is another important element that could reduce the solubility of calcium phosphate. There are several methods for evaluating demineralization. EDX analysis is a qualitative and quantitative demineralization determination method [51, 52]. Mutlu et al. found that remineralization was observed in all groups but homogeneous surface features were seen in group CPP-ACP with SEM/EDX analysis [51].The highest Ca rate was observed at the TM interface, likely attributable to CPP-ACP, and the RP surface showed the highest amount of F ratio because it was the only remineralizing agent that contained fluoride in this study. According to a study, EDX analysis provides only superficial information [53]. This study obtained elemental percentages using one sample from each group, but a more comprehensive analysis is required. The study is also limited by the fact that the in-vitro environment cannot perfectly emulate the oral environment, necessitating further clinical studies. Additionally, the study did not account for the impact of brushing on the remineralization agents, so the abrasion effect and the agents’ resistance to abrasion must be considered and added to the parameters and since the enamel defects in our study are limited to the outer half, studies on lesions of different depths are needed.

## Conclusion

Under the limitations of this in-vitro study, the results indicate that the use of remineralizing agents or resin infiltration methods alone or in combination did not result in any discernible differences. However, improvement of the enamel lesions was observed in all treatment procedures. The combined use of remineralization agents and resin infiltration did not alter the effectiveness of the treatment for initial enamel lesions. This study concluded that resin infiltration and remineralization agents, either in combination or alone, might be the optimal treatment for the onset of demineralization in the outer half of the enamel (DIAGNOdent Pen score 14–20).

## Data Availability

The data presented in this study are available on request from the corresponding author.
